# Prevalence of Problematic Digital Media Use Among Young Adults: Protocol for a Systematic Review

**DOI:** 10.2196/82245

**Published:** 2026-03-05

**Authors:** Rachelle Marie Chanmany Pastor, Fayette Nguyen Truax, Safiye Sahin, Janet Donnelly, Jan M Nick, Shanalee Tamares

**Affiliations:** 1 School of Nursing, Loma Linda University, 11262 Campus St., Loma Linda, CA, 92354, United States, 1 5622196101; 2 Sharp Health Care, San Diego, CA, United States; 3 JBI Center for Evidence Synthesis Loma Linda University Health, Loma Linda, CA, United States; 4 Loma Linda University Libraries, Loma Linda, CA, United States

**Keywords:** behavioral addiction, technology use disorder, screen time, digital addiction, internet overuse

## Abstract

**Background:**

Problematic digital media use (PDMU) among young people has been on the rise. PDMU is defined as excessive use of digital media, the internet, or electronic communication leading to user dysfunction and harm to other individuals. Evidence links excessive use of media with various mental health disorders, behavioral problems, substance abuse, poor sleep hygiene, and social dysfunction. This maladaptive behavior is pervasive among young people, yet there is a paucity of studies that comprehensively examine the phenomenon in this specific population.

**Objective:**

This systematic review seeks to examine the current global prevalence of PDMU among young adults aged 18 to 24 years, explore the extent of the issue across different regions of the world, and identify key factors contributing to its occurrence.

**Methods:**

The proposed systematic review will use the Prevalence Estimates Reviews – Systematic Review Methodology Group (PERSyst) methodology for systematic reviews of prevalence and incidence. A 3-step search strategy will be used covering 2020 to 2025, with no language restrictions. Nine sources will be searched: Embase (Elsevier), PubMed, PsycInfo (EBSCO), Web of Science Core Collection (Clarivate), Communication & Mass Media Complete (EBSCO), LILACS (VHL Search Portal), China National Knowledge Infrastructure, ProQuest Dissertations and Theses Global, and Google Scholar. Study selection will follow a 3-step process, including critical appraisal for methodological quality. Standardized data extraction tools will be used. Two reviewers will make decisions independently; conflicts will be resolved through consensus. Narrative synthesis will be conducted, and where possible, a meta-analysis will estimate PDMU prevalence with 95% CIs using a random-effects model. Heterogeneity will be assessed using the chi-square and *I*^2^ statistics. The Grading of Recommendations Assessment, Development, and Evaluation approach will be used to assess the certainty of the evidence.

**Results:**

The protocol was completed in July 2025. The comprehensive search of electronic bibliographic databases began in September 2025, followed by deduplication, screening, and selection of eligible studies. Title and abstract screening were completed in January 2026, and full-text review is ongoing. Data extraction and synthesis will be conducted between March and April 2026. The manuscript will be prepared and completed by August 2026, with plans to submit the manuscript for publication in September 2026.

**Conclusions:**

The planned review contributes to the growing body of evidence on digital media use among young adults by highlighting its potential impact on overall well-being. To our knowledge, this is the first comprehensive synthesis focused specifically on this population. The findings are expected to highlight the need for routine screening and early intervention strategies to address the social, mental, and physical health risks associated with digital media overuse in young adulthood.

## Introduction

### Background

Problematic digital media use (PDMU) among young people has been on the rise [1]. There is evidence suggesting that risks of maladaptive technological use may result in overuse and addiction [2]. PDMU is defined as excessive use of digital media, the internet, or electronic communication leading to user dysfunction and harm to other individuals [2,3]. While PDMU is primarily characterized by harm to the user, emerging literature indicates that its impacts may extend beyond the individual through relational disruption, impaired role functioning, and reduced interpersonal presence (eg, phubbing) [3-5]. The PDMU umbrella term encompasses several conceptualizations, such as internet gaming disorder (IGD) or problematic gaming, social media addiction (SMA) or problematic social media use, and problematic internet use (PIU) or internet addiction. Among youth, problematic use of media is linked to various mental health disorders, behavioral problems, substance abuse, poor sleep hygiene, and social dysfunction [2,6,7]. These health outcomes can negatively impact this population’s growth, development, and well-being, as well as their potential to lead active and productive adult lives.

While the body of evidence on PDMU is still emerging, this multifaceted issue is correlated with a multitude of psychological and behavioral conditions [2,3,7] affecting children and young people [8]. Given the ubiquity of digital media, specifically the internet, social media, and interactive gaming, concerns of overstimulation, distraction, and addiction are relevant as these media are easily accessed through handheld electronic devices, such as smartphones or tablets. Among young adults, such pervasive and continuous exposure (eg, gaming disorder) has been associated with difficulties in sustained attention, impaired prefrontal cortex functioning, diminished cognitive control, and potential disruptions in working memory processes [4,5]. However, the amount of time spent on digital media does not necessarily indicate dysfunctional use [9]. Instead, it is how the media is used—and its impact on well-being and daily life—that distinguishes acceptable, normal use from problematic use.

Researchers continue to examine related problematic technological issues, such as addiction to internet gaming [10,11], addiction to social media [12,13], and problematic use of the internet [2,14]. Addiction to gaming, or IGD, is described in the American Psychiatric Association’s *Diagnostic and Statistical Manual of Mental Disorders, Fifth Edition, Text Revision*. The World Health Organization (WHO) included IGD in the *International Classification of Diseases, 11th Revision*, and defines it as a “pattern of gaming behavior [‘digital-gaming’ or ‘video-gaming’] characterized by impaired control over gaming, increasing priority given to gaming over other activities to the extent that gaming takes precedence over other interests and daily activities, and continuation or escalation of gaming despite the occurrence of negative consequences” [15]. The latest systematic review on IGD, by Gao et al [16], showed that its prevalence was significantly higher among young adults than adolescents (10.4%, 95% CI 8.8%-11.9% vs 8.8%, 95% CI 7.5%-10%); however, the authors cautioned that their systematic review reflected post–COVID-19 activities, when there was a reduction in outdoor activities and increased risk of developing IGD. Indeed, several studies found significant increased incidence of IGD during the pandemic [2,15].

Another area of study in PDMU literature is SMA, described as individuals who experience addiction-like symptoms resulting from social media use, manifesting as extreme preoccupation and compulsion in social media platforms regardless of negative consequences [7]. Social media is highly popular and heavily used by young adults because this population is the first to grow up in an immensely digitalized society. Still, sustained engagement with social media is not without consequences. A recent study on college-aged students (N=571; mean age 23.61, SD 5.00 years) revealed that 22.7% were addicted to social media, scoring significantly higher on depressiveness, anxiety, and somatization than the group who was not addicted to social media [13]. Those addicted to social media were also found to exhibit higher use frequency, time spent, and perceived importance. These findings echo a recent systematic review’s findings that showed moderate but statistically significant correlations between SMA and depression, anxiety, and stress among young adults and adolescents [7]. Unlike IGD, SMA is currently not officially recognized and classified as an addiction or mental disorder in the *Diagnostic and Statistical Manual of Mental Disorders, Fifth Edition*, or *International Classification of Diseases, 11th Revision*, although research on this condition continues to expand.

PIU is a further subtype related to PDMU. PIU is an addictive behavioral condition characterized by repeated, uncontrollable online behavior that is risky, excessive, or impulsive, resulting in significant harm to the user [2,14]. Consequently, this behavior has been linked to physical and emotional functional impairments, relationship conflicts, compulsive internet use (ie, internet use that is difficult to control and continues despite negative effects on daily life), and mental health problems, such as depression. In a cross-cultural study (N=5130) examining PIU in 15 countries across Europe, South America, North America, and Asia, Lopez-Fernandez et al [14] found that emailing, social networking, shopping, and video streaming were the most common online activities prevalent among young adults with an average age of 24.71 (SD 8.70) years. The study participants tended to be single university students, and most used smartphones and computers or laptops, spending approximately 2 to 3 hours per day on online entertainment via computers and between 1 and 6 hours per day using smartphones. PIU frequency rates were high in English-speaking countries (ie, the United Kingdom and the United States); however, Asian countries were found to have the highest rates of PIU. Despite this, the researchers concluded that further research is needed with more representative samples for better generalization.

As research continues to expand on the divergent subtypes of PDMU [5], combining these subtypes into one comprehensive review provides a macrolevel view of the gravity of PDMU worldwide. Understanding the measured burden of this condition can inform the planning and development of targeted interventions aimed at mitigating risks associated with digital addiction. This maladaptive behavior is pervasive among young people, yet there is a paucity of studies that comprehensively synthesize the phenomenon in this specific population. The young adulthood period is characterized by high levels of digital media engagement and vulnerability to problematic use behaviors [17]. Limited studies on sociodemographic factors, such as age, gender, and socioeconomic status, show variable associations with PDMU [12-14]. Research on problematic smartphone and internet use has identified a broad range of psychological and behavioral predictors (eg, negative affectivity, fear of missing out, and psychological well-being), whereas sociodemographic factors alone appear less frequently as primary predictors in the literature on young adults [18]. Due to this paucity, a comprehensive systematic review and meta-analysis will be conducted with the aim of (1) examining the global prevalence of PDMU among young adults and (2) exploring the extent of the issue across different regions of the world while identifying key factors contributing to its occurrence. In this study, the term “young adult” is used for individuals aged 18 to 24 years, consistent with classifications used by the Centers for Disease Control and Prevention [19] and the WHO [20].

### Review Question

This review will address the following research questions:

1. What is the global prevalence of PDMU among young adults aged 18 to 24 years?
2. How does PDMU vary by age category, gender, country or region, educational level, and employment status?

## Methods

### Study Design

The proposed systematic review will be conducted in accordance with the methodology for systematic reviews of prevalence and incidence by the Prevalence Estimates Reviews – Systematic Review Methodology Group (PERSyst) [21]. The team used the PRISMA-P (Preferred Reporting Items for Systematic Reviews and Meta-Analyses Protocols) guidelines to prepare this protocol [22] and will use the PRISMA (Preferred Reporting Items for Systematic Reviews and Meta-Analyses) extension for abstracts [22] and the PRISMA 2020 flow diagram to show the results of the search and screening phases [23]. These actions are planned to ensure completeness in meeting the standards for systematic reviews.

### Eligibility Criteria

This proposed systematic review uses the elements of the condition, context, and population framework as outlined in the Joanna Briggs Institute (JBI) Manual for Evidence Synthesis [24]. Summarized points are shown in Table 1.

**Table 1.** Inclusion and exclusion criteria.

	Inclusion criteria	Exclusion criteria
Participants	Young adults aged 18 to 24 years Male, female, and nonbinary participants Any sociodemographic characteristics	Age <18 or >24 years Electronic screen dependence due to speech or motor impairments
Condition	Problematic use of smartphones, social media, online gaming, and the internet Self-report instruments (ie, IAT^a^, PIUQ^b^, SMD^c^, or GAS^d^, as well as other tools) Clinician diagnostic documentation (ie, *DSM-5*^e^ [eg, for internet gaming disorder]; *ICD-11*^f^ [eg, for gaming disorder]; clinical interviews that explore patterns of functional impairment, withdrawal, and loss of control associated with digital media use; and the SCID^g^ or similar validated diagnostic interviews)	Studies reporting general or positive digital media use
Context	Global Diverse settings, such as the home, the clinical setting, health care, the educational setting, the workplace, and community-based settings All populations (the general population and marginalized populations [ie, homeless individuals, refugees, or those in transitional housing])	Studies that focus on correctional or forensic institutions (eg, prisons or juvenile detention centers) and rehabilitation or detoxification centers
Type of study	Analytical observational studies Descriptive observational studies Experimental designs that report prevalence at baseline or the screening or eligibility phase Conference abstracts	Qualitative studies Case reports Case series Editorials and commentaries Letters to the editor Protocols Opinion pieces Synthesis reviews Literature reviews
Search limits	Any language Last 5 years (2020-2025)	None

a IAT: Internet Addiction Test.

b PIUQ: Problematic Internet Use Questionnaire.

c SMD: Social Media Disorder Scale.

d GAS: Gaming Addiction Scale.

e *DSM-5*: *Diagnostic and Statistical Manual of Mental Disorders, Fifth Edition*.

f *ICD-11*: *International Classification of Diseases, 11th Revision*.

g SCID: Structured Clinical Interview for DSM-5*.*

### Search Strategy

The search strategy will aim to locate both published and unpublished studies. A 3-step search strategy will be used in this review. First, an initial search of Embase (Elsevier) was undertaken by a library scientist to identify articles on the topic. The text words contained in the titles and abstracts of relevant articles, the index terms used to describe the articles, and the synonyms suggested by this database will be used to develop the full search strategy (Multimedia Appendix 1). Studies published in any language and from 2020 to 2025 will be included to determine the currency of prevalence rates and trends of prevalence over time. Recent data account for the rapidly evolving technology ecosystems and reflect the current social media landscapes necessary for the recommendation of up-to-date and evidence-based interventions. Another library scientist will independently peer review the search strategy for completeness and rigor, adhering to the PRESS (Peer Review of Electronic Search Strategies) guidelines [25].

The second step of the search strategy will adapt the identified keywords and index terms from the initial search to suit the specific indexing and search functionalities of each included database and/or information source, which will comprise Embase (Elsevier), PubMed, PsycInfo (EBSCO), Web of Science Core Collection (Clarivate), Communication & Mass Media Complete (EBSCO), LILACS (VHL Search Portal), China National Knowledge Infrastructure, and ProQuest Dissertations and Theses Global. Additionally, Google Scholar will be searched, and only the first 200 results will be considered as recommended [26].

The third stage of the search strategy will involve both backward and forward citation checking. Specifically, the reference lists of all included sources of evidence will be systematically screened to identify additional relevant studies. For forward citation checking, citation-tracking functionalities within search platforms, such as Web of Science and Google Scholar will be used to identify more recent articles that have cited our included studies. Comprehensive documentation of all search processes and reporting of results will adhere to the PRISMA-S (PRISMA extension for reporting literature searches in systematic reviews) statement, thereby ensuring the transparency, reproducibility, and comprehensiveness of the reporting details of the search methodology [27].

### Study Screening and Selection

Following the 3-step search, all identified citations will be collated and uploaded into EndNote (version 21; Clarivate Analytics), and duplicates will be removed. After deduplication, potentially relevant studies will be retrieved, and their citation details will be imported into the JBI System for the Unified Management, Assessment, and Review of Information. Study screening and selection will follow a 3-step process to increase specificity and sensitivity.

First, we will carry out two actions to minimize title and abstract screening errors: (1) creating a 1-page summary of inclusion and exclusion criteria to use while voting and (2) conducting a pilot test of 10 titles and abstracts to calibrate clarity of understanding of the inclusion and exclusion of participants, concept, context, and types of studies. Any combination of 2 team members will review titles and abstracts for assessment against the inclusion criteria for the review.

In the second step, the full texts of reports of titles included from the previous step will be collected and reviewed. In the case of any studies published in languages other than English, they will be translated using the large language model DeepL Translate (DeepL SE); this artificial intelligence program has been shown to be highly accurate in translating scientific reports [28]. The full texts of selected reports will be assessed in detail against the inclusion criteria by 2 independent reviewers. The full citations and reasons for exclusion of papers that do not meet the inclusion criteria at the full-text step will be recorded and reported in the systematic review. Reasons for exclusion will include ineligible participants, condition, or context; ineligible study type; duplicate study; unavailability through interlibrary loan; or unavailability due to high costs, among others.

For the third step of screening, included studies from the previous step will undergo assessment of methodological quality using the JBI’s critical appraisal tools [29]. Two independent reviewers will conduct the critical appraisal to determine the final studies included.

In each stage of the selection process, conflicts in voting between assessors will be reviewed and resolved through discussion or with one or more additional reviewers. The results of the search and the study inclusion process will be reported in full in the final systematic review and presented in a PRISMA flow diagram [23].

### Assessment of Methodological Quality

Two or more independent reviewers will conduct a critical appraisal of the methodological quality of the included studies in this proposed systematic review using the standardized critical appraisal instrument from the JBI for prevalence studies. This critical appraisal tool is specifically designed to evaluate a study’s methodological merit regardless of whether the design is cross-sectional or cohort based [24].

During critical appraisal, studies will be assessed using 3 grades: high risk (0%-33% of the criteria met), medium risk (34%-66% of the criteria met), and low risk (>67% of the criteria met). Studies that do not meet a certain quality threshold will be considered as having a high risk of bias and will be excluded from the extraction and synthesis phases. Studies with medium or low risk of bias will be included. Disagreements will be resolved through discussion or with a third reviewer. Results will be reported in a narrative format and risk-of-bias tables for each study design.

### Data Extraction

To standardize the data extraction process, the team will use the JBI System for the Unified Management, Assessment, and Review of Information standardized tool to extract data [30]. The extraction template will capture key information on each study, such as (1) bibliometric characteristics, including country, year of publication, time frame for data collection, and language of publication; (2) participant characteristics, such as age, gender, ethnicity, level of education, population density (urban or rural), and other socioeconomic factors (additional characteristics may include mental health status, sleep patterns, personality traits, substance use, living situation, relationship status, digital media use habits [eg, screen time and type of media], and academic or employment status when available); (3) conditions and measurement methods used, such as study design, name of validated tools used to collect data, and type of digital device; and (4) description of the main results, including sample size, number of cases, prevalence, and CIs.

During extraction, any modifications to the tools will be detailed and justified in the narrative of the full review. To ensure consistency, the team will complete in-person training by extracting data from 2 studies together and comparing results. Once training has validated the skill level of the team, any 2 trained team members will extract the data. Any disagreements that arise between the reviewers will be resolved through discussion or with a third reviewer. In cases in which key information is missing, unclear, or contradictory, study authors will be contacted for clarification. If there is no response, data that remain missing or unclearly reported will be noted as “NR” (not reported) in the data extraction form.

### Data Synthesis

Study data will, where possible, be pooled in a proportional meta-analysis following the PERSyst guidelines for processing, reporting, and interpretation [21]. The aim of this specific type of meta-analysis will be to pool study results, improve statistical power, and provide guidance on the global prevalence of PDMU. Results can provide evidence for clinicians, researchers, educators, and policymakers. The meta-analysis will be conducted using the PERSyst MA software (version 1.0) based on the R *meta* package (version 7.00; R Foundation for Statistical Computing) [31], and it will be organized following a 3-step approach.

First, outcome data will be standardized to a common effect estimate to ensure consistency. Two reviewers will cross-check the extracted data on prevalence for accuracy and consistency; variations in population labels and definitions of prevalence will be coordinated across studies to improve comparability. As our intention with the meta-analysis is to generalize the pooled prevalence estimate, we will use a random-effects model if there are 5 or more studies included. This is based on guidance by Tufanaru et al [32]. Statistical parameters will be input into the model using the Freeman-Tukey double arcsine transformation to stabilize variance in prevalence estimates. For fewer than 5 studies, a fixed-effects model will be used, and statistical heterogeneity will be checked. If heterogeneity is lower than 50%, the fixed-effects model will be appropriate. However, when heterogeneity is higher than 50%, a narrative approach would be preferred [33,34].

Second, we will conduct the meta-analyses using the selected model and use forest plots to display the distribution of individual and pooled prevalence estimates, corresponding 95% CIs, and measures of heterogeneity. To aid in interpretation, we will express results using percentages [35]. Outliers from the study results will be rechecked for coding errors. If the number of studies is small, we will prioritize point estimates, CIs, and the pooled estimate rather than relying on the chi-square test (Cochran *Q* statistic) for heterogeneity. The *I*^2^ statistic will quantify heterogeneity, categorized as low (<25%), moderate (25%‐75%), or high (>75%) [33,34].

Third, the team will conduct post hoc analyses for sensitivity, subgroup performance, and publication bias. Sensitivity analysis will use the 1-study removal method to assess individual studies’ impact on the pooled estimate. Subgroup analyses will explore heterogeneity when possible by stratifying data across different subtypes of PDMU, WHO’s 6 geographic regions [36], settings, sample size, diagnostic tools used, and other important bibliometric and participant characteristics. If at least 10 studies are included, publication bias will be assessed using funnel plots in the Comprehensive Meta-Analysis software (version 3.3.070; Biostat, Inc), with asymmetry tested via the Egger test.

If meta-analysis is not possible due to too much variability in the extracted data or too few included studies, the findings will be presented in narrative format, tables, figures, and graphical displays to summarize the evidence and study characteristics descriptively.

## Results

The study aims to examine the global prevalence and key factors of PDMU among young adults as this population is at risk of maladaptive behaviors and poor psychosocial health outcomes and underrepresented in the literature. The protocol has been registered in PROSPERO (registration CRD420251075912). The following databases were initially searched in April 2025: PROSPERO, MEDLINE, the Cochrane Database of Systematic Reviews, and *JBI Evidence Synthesis*, and no ongoing systematic reviews of prevalence on the topic were identified. The preliminary search yielded 1189 primary studies, suggesting sufficient primary research for the planned synthesis research.

The comprehensive search began in September 2025, with screening and selection of eligible studies following a 3-step search process. The process involved searching electronic bibliographic databases or information sources: Embase (Elsevier), PubMed, PsycInfo (EBSCO), Web of Science Core Collection (Clarivate), Communication & Mass Media Complete (EBSCO), LILACS (VHL Search Portal), China National Knowledge Infrastructure, ProQuest Dissertations and Theses Global, and Google Scholar. Once the search was completed, and after deduplication, a total of 2113 results were retrieved from the databases for title and abstract screening. The team began title and abstract screening in October 2025 using a 1-page summary of inclusion and exclusion criteria for voting. Conflicts were reviewed and resolved through discussion or with one or more additional reviewers. This phase of screening lasted until the beginning of January 2026. Subsequently, the team began reviewing the full texts of 1096 reports of titles included from the previous step. The full texts of selected reports are being assessed in detail against the inclusion criteria by 2 independent reviewers. We will provide a list of excluded studies from full-text screening in the form of a supplementary file. No deviations from the registered protocol have occurred.

To illustrate the inclusion process, a PRISMA flow diagram will be included. Additionally, results from the comprehensive review will be presented in tables to show general findings, risk of bias, and quality of the studies. Data extraction and synthesis will be conducted between March and April 2026. Subsequently, the manuscript will be prepared and is expected to be completed by the end of August 2026. We plan to submit the manuscript for publication in September 2026. Finally, to bring awareness of this pervasive issue, findings from the study will be disseminated at relevant conferences and shared with stakeholders, such as health care providers, educators, researchers, and public health professionals.

## Discussion

### Expected Findings

This protocol presents a plan for the synthesis of the existing literature on PDMU among young adults, encompassing related constructs such as PIU, IGD, and SMA. The review will identify a wide range of social media platforms and online games specifically designed to maximize user engagement, which may contribute to adverse health outcomes. Reported consequences of excessive digital media use include increased alcohol consumption, sleep disturbances, and mental health issues [2]. Additionally, overlapping demographic and health-related patterns have been observed across PIU, IGD, and SMA. While substantial evidence exists linking digital media overuse to negative physical and mental health outcomes in school-aged populations, comparatively little is known about its effects on young adults. This gap is likely due to limited research attention directed toward this age group. Consequently, the young adult population was selected for this review due to its underrepresentation in the literature and the need for a more comprehensive understanding of how digital media use affects their health and well-being.

PDMU among young adults has reached increasingly concerning levels in recent years. However, research on screen time behaviors in this population remains limited, potentially due to their relatively infrequent engagement with health care providers. Despite this gap, a growing body of literature has documented the adverse physical and mental health outcomes associated with excessive media use in young adults [37]. For example, a recent systematic review by Brautsch et al [38] identified significant associations between digital media use and sleep onset latency, sleep duration, early awakening, sleep disturbances, daytime fatigue and dysfunction, sleep deficits, and overall sleep quality. These findings demonstrate that increased digital media use is consistently linked to poor sleep outcomes. Despite the professional guidelines and mounting evidence highlighting the risks of PDMU, many young adults continue to disregard recommended practices for healthy media consumption in their daily routines.

A recent survey conducted as part of the Millenium Cohort Study in the United Kingdom found that 48% of young adults (aged 18-24 years) self-identified as being “addicted” to social media. This finding is concerning as perceived addiction to digital platforms may signal long-term risks to the user’s mental and physical well-being. Additionally, an online cross-sectional survey conducted during the COVID-19 pandemic examined screen time behaviors among young adults (aged 18-28 years). The study found that time spent on specific screen-based activities, particularly social media and entertainment, was associated with increased symptoms of depression, anxiety, psychological distress, and substance abuse [39]. These findings raise significant public health concerns as the long-term health effects of PDMU are not yet well established given that research on this condition is still emerging. A comprehensive investigation into this issue is timely and necessary given the rapid advancement of digital technologies and their pervasive integration into the daily lives of young adults. Although prior studies have explored aspects of this phenomenon, the existing literature remains limited, particularly with respect to this age group.

### Strengths and Limitations

The strengths of this protocol methodology include a rigorous approach to creating a comprehensive, unbiased, reproducible, and transparent product while identifying research gaps and providing evidence-based recommendations. First, quantitative designs enable objective measurement of digital media behaviors and associated psychological outcomes using validated scales and rigorous statistical analyses. This approach enhances the reliability and generalizability of the findings across diverse populations. However, quantitative methods also present limitations. They are typically less effective in capturing the contextual and subjective nuances of user experiences, have a limited ability to explore a topic, and often face challenges in establishing causality due to their cross-sectional or observational nature. Additionally, these studies may be subject to recall bias and social desirability bias, particularly when relying on self-reported data. Finally, the possibility of reporting bias and selective publication bias may lead to conflated findings of prevalence.

While a comprehensive search strategy will be used, relevant studies could be overlooked. Furthermore, despite using predefined criteria, judgments about the relevance and quality of the articles involve some level of subjectivity from the study team members. The completion of this systematic review protocol on PDMU is essential to ensure transparency, uphold methodological rigor, and maintain research integrity. Given the rapid evolution of digital media use and its psychosocial impacts, publishing a protocol ensures that the review follows best practices and meets the standards required for evidence-based policymaking and future research.

### Conclusions and Recommendations

This review will add to the growing body of evidence on PDMU among young adults by emphasizing its potential impact on their overall well-being. To the best of our knowledge, this is the first comprehensive synthesis focused specifically on this age group. The findings will underscore the importance of implementing routine screening and early intervention strategies to mitigate the social, psychological, and physical health risks associated with digital media overuse in young adulthood.

Future research should prioritize interventional studies aimed at reducing digital media use among young adults while also providing evidence-based recommendations for achieving a balanced integration of screen time and green time in their daily routines.

## Supplementary material

10.2196/82245 Multimedia Appendix 1 Search strategy by database and platform, date searched, and results.

10.2196/82245 Checklist 1 PRISMA-P 2015 checklist.

## Abbreviations

IGD: internet gaming disorder
JBI: Joanna Briggs Institute
PDMU: problematic digital media use
PERSyst: Prevalence Estimates Reviews – Systematic Review Methodology Group
PIU: problematic internet use
PRESS: Peer Review of Electronic Search Strategies
PRISMA: Preferred Reporting Items for Systematic Reviews and Meta-Analyses
PRISMA-P: Preferred Reporting Items for Systematic Reviews and Meta-Analyses Protocols
PRISMA-S: Preferred Reporting Items for Systematic Reviews and Meta-Analyses extension for reporting literature searches in systematic reviews
SMA: social media addiction
WHO: World Health Organization
