# Supplementary material for: Prevalence of Problematic Digital Media Use Among Young Adults: Protocol for a Systematic Review
Source: JMIR Res Protoc. 2026 Mar 5;15:e82245. doi: 10.2196/82245 (PMC12978971; doi:10.2196/82245)
Supplement: Multimedia Appendix 1 [file resprot-v15-e82245-s001.docx]

# Appendices

### Appendix I: Search strategy database and platform, date searched and the results.

Embase (Elsevier platform)

Search conducted: June 11, 2025

| **Search #** | **Query** | **Records retrieved** |
| --- | --- | --- |
| S1 | 'computer addiction'/exp OR 'compulsive computer use':ti,ab,kw OR 'computer addiction':ti,ab,kw OR 'computer-related addiction':ti,ab,kw OR 'cyber-addiction':ti,ab,kw OR 'cyberaddiction':ti,ab,kw OR 'digital addiction':ti,ab,kw OR 'electronic media overuse':ti,ab,kw OR 'media addiction':ti,ab,kw OR 'pathological computer use':ti,ab,kw OR 'pathological media use':ti,ab,kw OR 'problematic computer use':ti,ab,kw OR 'problematic cyber use':ti,ab,kw OR 'problematic digital media use':ti,ab,kw OR 'problematic media use':ti,ab,kw OR 'screen media overuse':ti,ab,kw OR 'tablet addiction':ti,ab,kw OR 'technology addiction':ti,ab,kw OR 'game addiction'/exp OR 'compulsive gaming':ti,ab,kw OR 'computer game addiction':ti,ab,kw OR 'excessive gaming':ti,ab,kw OR 'game addiction':ti,ab,kw OR 'gaming addiction':ti,ab,kw OR 'gaming dependency':ti,ab,kw OR 'gaming disorder':ti,ab,kw OR 'internet gaming disorder':ti,ab,kw OR 'on-line game addiction':ti,ab,kw OR 'online game addiction':ti,ab,kw OR 'online gaming addiction':ti,ab,kw OR 'pathological computer gaming':ti,ab,kw OR 'pathological gaming':ti,ab,kw OR 'pathological internet gaming':ti,ab,kw OR 'pathological video gaming':ti,ab,kw OR 'problematic computer gaming':ti,ab,kw OR 'problematic digital gaming':ti,ab,kw OR 'problematic gaming':ti,ab,kw OR 'problematic internet gaming':ti,ab,kw OR 'problematic mobile gaming':ti,ab,kw OR 'problematic online gaming':ti,ab,kw OR 'problematic video gaming':ti,ab,kw OR 'problematic videogaming':ti,ab,kw OR 'video game addiction':ti,ab,kw OR 'videogame addiction':ti,ab,kw OR 'social media addiction'/exp OR 'pathological social media use':ti,ab,kw OR 'problematic social media use':ti,ab,kw OR 'social media addiction':ti,ab,kw OR 'social media disorder':ti,ab,kw OR 'social network site addiction':ti,ab,kw | 10,641 |
| S2 | (('problematic':ti,ab,kw OR 'disorder':ti,ab,kw OR 'addiction':ti,ab,kw OR 'excessive':ti,ab,kw OR 'compulsive':ti,ab,kw OR 'dependence':ti,ab,kw OR 'dependency':ti,ab,kw OR 'maladaptive':ti,ab,kw OR 'unhealthy':ti,ab,kw OR 'abusive':ti,ab,kw OR 'dysfunctional':ti,ab,kw OR 'intense':ti,ab,kw OR 'overuse':ti,ab,kw OR 'pathological':ti,ab,kw OR 'risky':ti,ab,kw) AND ('digital media use':ti,ab,kw OR 'digital media usage':ti,ab,kw OR 'internet use':ti,ab,kw OR 'internet usage':ti,ab,kw OR 'social media use':ti,ab,kw OR 'social media usage':ti,ab,kw OR 'internet gaming use':ti,ab,kw OR 'internet gaming usage':ti,ab,kw OR 'smartphone use':ti,ab,kw OR 'smartphone usage':ti,ab,kw OR 'video game use':ti,ab,kw OR 'video game usage':ti,ab,kw OR 'technology use':ti,ab,kw OR 'technology usage':ti,ab,kw OR 'computer use':ti,ab,kw OR 'computer usage':ti,ab,kw OR 'computer related':ti,ab,kw OR 'cyber use':ti,ab,kw OR 'cyber usage':ti,ab,kw OR 'screen media':ti,ab,kw OR 'tablet use':ti,ab,kw OR 'tablet usage':ti,ab,kw OR 'gaming':ti,ab,kw OR 'videogaming':ti,ab,kw)) | 9,943 |
| S3 | (('observational study'/exp OR 'non experimental studies':ti,ab,kw OR 'non experimental study':ti,ab,kw OR 'nonexperimental studies':ti,ab,kw OR 'nonexperimental study':ti,ab,kw OR 'observation studies':ti,ab,kw OR 'observation study':ti,ab,kw OR 'observational studies':ti,ab,kw OR 'observational studies as topic':ti,ab,kw OR 'observational study':ti,ab,kw OR 'observational study as topic':ti,ab,kw OR 'national longitudinal study of adolescent health'/exp OR 'prevalence'/exp OR 'prevalence study':ti,ab,kw OR 'cohort studies':ti,ab,kw OR 'cohort study':ti,ab,kw OR 'cross-sectional study'/exp OR 'cross-sectional design':ti,ab,kw OR 'cross-sectional research':ti,ab,kw OR 'cross-sectional studies':ti,ab,kw OR 'cross-sectional study':ti,ab,kw OR 'descriptive study':ti,ab,kw OR 'registry data':ti,ab,kw OR 'census data':ti,ab,kw OR 'population data'/exp OR 'population data':ti,ab,kw) OR (('experimental study'/exp OR 'experimental studies':ti,ab,kw OR 'experimental study':ti,ab,kw OR 'randomized controlled trial'/exp OR 'controlled trial, randomized':ti,ab,kw OR 'randomised controlled study':ti,ab,kw OR 'randomised controlled trial':ti,ab,kw OR 'randomized controlled study':ti,ab,kw OR 'randomized controlled trial':ti,ab,kw OR 'trial, randomized controlled':ti,ab,kw OR 'clinical trial'/exp OR 'clinical drug trial':ti,ab,kw OR 'clinical trial':ti,ab,kw OR 'major clinical trial':ti,ab,kw OR 'trial, clinical':ti,ab,kw OR 'intervention study'/exp OR 'intervention studies':ti,ab,kw OR 'intervention study':ti,ab,kw OR 'intervention trial':ti,ab,kw OR 'interventional studies':ti,ab,kw OR 'interventional study':ti,ab,kw OR 'interventional trial':ti,ab,kw OR 'conference abstract'/exp OR 'conference abstract':ti,ab,kw) AND ('prevalence'/exp OR 'prevalence':ti,ab,kw OR 'prevalence study':ti,ab,kw OR 'incidence'/exp OR 'incidence':ti,ab,kw OR 'incidence rate':ti,ab,kw OR 'rate, incidence':ti,ab,kw))) | 3,187,632 |
| S4 | ('young adult'/exp OR 'adult, young':ti,ab,kw OR 'prime adult':ti,ab,kw OR 'prime adults':ti,ab,kw OR 'young adult':ti,ab,kw OR 'young adults':ti,ab,kw OR 'young adulthood':ti,ab,kw OR 'college aged':ti,ab,kw OR ((('18' OR '19' OR '20' OR '21' OR '22' OR '23' OR '24') NEAR/2 ('year' OR 'years' OR 'age' OR 'aged' OR 'ages')):ab)) | 1,820,443 |
| S5 | (2020:py OR 2021:py OR 2022:py OR 2023:py OR 2024:py OR 2025:py) | 10,267,921 |
| S6 | (#1 OR #2) AND #3 AND #4 AND #5 | 1,189 |
